# Update to the study protocol, including statistical analysis plan for a randomized clinical trial comparing comprehensive cardiac rehabilitation after heart valve surgery with control: the CopenHeart_VR_ trial

**DOI:** 10.1186/s13063-015-0562-z

**Published:** 2015-02-05

**Authors:** Kirstine Laerum Sibilitz, Selina Kikkenborg Berg, Tina Birgitte Hansen, Signe Stelling Risom, Trine Bernholdt Rasmussen, Christian Hassager, Lars Køber, Christian Gluud, Lau Caspar Thygesen, Jane Lindschou, Jean Paul Schmid, Rod S Taylor, Ann-Dorthe Zwisler

**Affiliations:** The Heart Centre, Department of Cardiology, Rigshospitalet, Copenhagen University Hospital, Blegdamsvej 9, 2100 Copenhagen, Denmark; Department of Cardiology, Gentofte Hospital, Gentofte, Denmark; National Institute of Public Health, University of Southern Denmark, Copenhagen, Denmark; Department of Cardiology, Roskilde Hospital, Roskilde, Denmark; Copenhagen Trial Unit, Centre for Clinical Intervention Research, Rigshospitalet, Copenhagen University Hospital, Copenhagen, Denmark; Cardiology Clinic, Tiefenau Hospital and University of Bern, Bern, Switzerland; University of Exeter Medical School, Health Services Research, University of Exeter, Exeter, Devon UK; Department of Cardiology, Holbæk Sygehus, Holbæk, Denmark

**Keywords:** Heart valve surgery, Rehabilitation, Physical exercise, Psycho-education

## Abstract

**Background:**

Heart valve diseases are common with an estimated prevalence of 2.5% in the Western world. The number is rising because of an ageing population. Once symptomatic, heart valve diseases are potentially lethal, and heavily influence daily living and quality of life. Surgical treatment, either valve replacement or repair, remains the treatment of choice. However, post-surgery, the transition to daily living may become a physical, mental and social challenge. We hypothesize that a comprehensive cardiac rehabilitation program can improve physical capacity and self-assessed mental health and reduce hospitalization and healthcare costs after heart valve surgery.

**Methods:**

This randomized clinical trial, CopenHeart_VR_, aims to investigate whether cardiac rehabilitation in addition to usual care is superior to treatment as usual after heart valve surgery. The trial will randomly allocate 210 patients 1:1 to an intervention or a control group, using central randomization, and blinded outcome assessment and statistical analyses. The intervention consists of 12 weeks of physical exercise and a psycho-educational intervention comprising five consultations. The primary outcome is peak oxygen uptake (VO_2_ peak) measured by cardiopulmonary exercise testing with ventilatory gas analysis. The secondary outcome is self-assessed mental health measured by the standardized questionnaire Short Form-36. Long-term healthcare utilization and mortality as well as biochemistry, echocardiography and cost-benefit will be assessed. A mixed-method design will be used to evaluate qualitative and quantitative findings, encompassing a survey-based study before the trial and a qualitative pre- and post-intervention study.

**Conclusion:**

This randomized clinical trial will contribute with evidence of whether cardiac rehabilitation should be provided after heart valve surgery. The study is approved by the local regional Research Ethics Committee (H-1-2011-157), and the Danish Data Protection Agency (j.nr. 2007-58-0015).

**Trial registration:**

Trial registered 16 March 2012; ClinicalTrials.gov (NCT01558765).

## Update

This update relates to the CopenHeart_VR_ trial study protocol, a randomized clinical trial comparing comprehensive cardiac rehabilitation after heart valve surgery, including physical exercise and psycho-education, with usual care. This update should be read in conjunction with the original protocol publication [1].

### Revised trial population

The plan for this trial was to include patients having either open heart surgery or percutaneous heart valve procedures. However, because of administrative reasons and other local ongoing clinical trials including patients having percutaneous procedures, we were only able to include patients having open heart surgery.

### Statistical analysis plan

#### Updated sample size and power

Our original recruitment target was 210 with the recruitment period defined as two years. At the end of the two-year recruitment period in May 2014, we were only able to include 147 patients. Therefore, the power calculation has been repeated, based on this revised sample size.

The primary outcome is the continuous variable peak oxygen uptake (VO_2_ peak) measured by cardiopulmonary exercise testing. Based on the assumptions defined in the protocol (a minimum clinical meaningful difference of 3 mL/kg/min and standard deviation of 6 ml/kg/min), a sample size of 147 would provide 86% power to be able to reject the null hypothesis, with a type I error of 5%. The secondary outcome measure is mental health measured on the continuous variable mental health component scale on the Short Form-36 questionnaire. Based on the assumed minimum important difference of seven points and an updated standard deviation of nine points [2], we will be able to reject the null hypothesis with a probability of 99% and a type I error probability of 5%.

### Statistical analysis plan for the primary and secondary outcomes

While the primary analysis essentially remains the same as defined in the protocol, we propose slight revisions to the analysis model and handling of missing data, and also provide further specification of the handling of multiplicity of outcome testing.

#### Primary analysis

The level of significance is set at 5%. The intention-to-treat analysis using a mixed model with repeated measures (MMRM) for continuous outcome measures will be adjusted by the trial stratification variable ‘left ventricular ejection fraction <45%, yes/no’ [3].

#### Missing values and sensitivity analyses

As stated in the protocol, using MMRM ensures that missing data values will not create bias, as long as the values are missing at random [4]. For the primary and secondary outcomes, if a statistically significant result is obtained (*P* ≤0.05), we will undertake sensitivity analyses that use the method of multiple imputation [5,6] and also undertake imputation that assumes not missing at random and includes ‘worst-case’ and ‘best-case’ scenarios.

#### Multiplicity

The primary and secondary outcomes (VO_2_ peak and mental health component scale of Short Form-36) will be analyzed as stated above. The various exploratory outcome measures pre-defined in the protocol [1] will be analyzed with no adjustment of *P*-values due to multiplicity. Instead, the interpretation of each exploratory outcome measure will be assessed in the light of multiple testing; that is, statistically significant effects will be interpreted in the context of increased risk of type I error.

### Per-protocol analysis

In addition to the primary analysis, we will also undertake a per-protocol analysis to account for the variable adherence to the prescribed rehabilitation program by intervention group participants [7]. We expect that the effect of the intervention will depend on the participants’ adherence to the study protocol [8].

The per-protocol definition is based on the intervention consisting of two elements: physical exercise and a psycho-educational intervention. Adherence to both elements of the intervention will be described according to compliance data for self-reported training diaries, data from pulse watches, and records made in relation to the nurse consultations.

### Physical exercise

The physical exercise comprises three weekly sessions conducted between one and four months after surgery, a total of 36 sessions. Participation in the intervention will be defined as participation in an individual consultation to plan exercise training, receiving instruction in the use of the training diary and pulse watch, and participation in 75% (≥27) of the exercise sessions.

### Psycho-educational intervention

The psycho-educational intervention is based on the theories of RR Parse [9] and comprises one monthly consultation conducted within the first six months after surgery, a total of five consultations. Participation in the intervention will be defined as attending at least 80% (four out of five) of the psycho-educational consultations.

### Ethical approval

The study was approved by the regional Danish Research Ethics Committee in the Capital Region of Denmark (H-1-2011-157) and the Danish Data Protection Agency (j.nr. 2007-58-0015). All participants have given full written and oral informed consent.

## Abbreviation

MMRM: Mixed model with repeated measures
